# Barriers and facilitators of the effective use of DHIS2 data to improve program planning and monitoring in Uganda: a sequential mixed methods study

**DOI:** 10.1093/oodh/oqag002

**Published:** 2026-01-14

**Authors:** Suzanne N Kiwanuka, Steven N Kabwama, Noel Namuhani, Suruchi Gupta, Erica Layer, Patricia Mechael, Dustin G Gibson, Smisha Agarwal

**Affiliations:** Department of Health Policy Planning and Management, Makerere University School of Public Health, New Mulago Hill Road, Mulago, Kampala 10218, Uganda; Department of Community Health and Behavioral Science, Makerere University School of Public Health, New Mulago Hill Road, Mulago Kampala 10218, Uganda; Department of Global Public Health, Karolinska Institutet, Solna, Solnavägen 1E, floor 6, Stockholm Sweden; Department of Health Policy Planning and Management, Makerere University School of Public Health, New Mulago Hill Road, Mulago, Kampala 10218, Uganda; Center for Global Digital Health and Innovation, Department of International Health, Johns Hopkins University, 615 N. Wolfe Street, Baltimore, MD 21218, United States; Department of International Health, Johns Hopkins Bloomberg School of Public Health, 615 N. Wolfe Street, Baltimore, MD 21218, United States; health.enabled, Global Development Incubator, Washington, 1401 K Street NW, Suite 900 Washington DC 20005, United States; Center for Global Digital Health and Innovation, Department of International Health, Johns Hopkins University, 615 N. Wolfe Street, Baltimore, MD 21218, United States; Department of International Health, Johns Hopkins Bloomberg School of Public Health, 615 N. Wolfe Street, Baltimore, MD 21218, United States; health.enabled, Global Development Incubator, Washington, 1401 K Street NW, Suite 900 Washington DC 20005, United States; Center for Global Digital Health and Innovation, Department of International Health, Johns Hopkins University, 615 N. Wolfe Street, Baltimore, MD 21218, United States; Department of International Health, Johns Hopkins Bloomberg School of Public Health, 615 N. Wolfe Street, Baltimore, MD 21218, United States; Center for Global Digital Health and Innovation, Department of International Health, Johns Hopkins University, 615 N. Wolfe Street, Baltimore, MD 21218, United States; Department of International Health, Johns Hopkins Bloomberg School of Public Health, 615 N. Wolfe Street, Baltimore, MD 21218, United States

**Keywords:** DHIS2, digital health, data quality, data use for decision-making, HTM, immunization

## Abstract

Uganda adopted the District Health Information Software 2 (DHIS2) in 2012 to monitor public health programs. More than a decade later, the factors that facilitate or hinder the adoption and use of DHIS2 have not been well documented. This study uses a mixed methods design to understand these factors. Ugandan districts were categorized into high, medium and low performance based on weekly reporting, timeliness and completeness into DHIS2. Two districts were selected across each category of high (Yumbe and Maracha), moderate (Kakumiro and Budaka) and low performers (Jinja and Buikwe). In-depth interviews were conducted at national, district, and health facilities with program (immunization, HIV/AIDS, TB, and malaria) managers, district biostatisticians, district health officers, planners, and health facility managers. Data were analyzed thematically. Across the selected districts, the average adoption score (average of all three indicator scores listed above) ranged from 97% for high adopters to 60.3% for low adopters. Enablers of DHIS2 adoption included strong digital infrastructure, adequate and competent human resources, support from implementing partners, and financial incentives. Barriers to DHIS2 adoption were infrastructural challenges and system errors, including a lack of access rights to key cadres and server breakdown. Human resource limitations were also important barriers. Despite DHIS2’s role in assessing program performance and real time monitoring of campaigns, data use for decision making remains limited. Findings indicate that the limited investment in its operationalization impedes effective use. This implies improving in-country ownership to routinely upgrade equipment, provide reliable network, and recruit trained personnel.

## Introduction

The routine use and management of information at different levels is one of the essential components of the health system [1, 2]. Persistent challenges in health information systems and the lack of quality data hampers the measurement of progress and hinders timely decisions towards program improvement. In Uganda, fragmented health information systems and the limited use of electronic health records for clinical care and decision-making have posed several health systems challenges. To alleviate these, the Ministry of Health in Uganda launched the Health Information and Digital Strategic Plan 2020/2021 to 2024–2025 aimed at strengthening the health information system in the country [3].

The plan seeks to address data quality and late reporting in health facilities across the country by institutionalizing the use of patient-level digital systems at the point of care. It strategizes ensuring access to high quality health data at national and sub-national levels and enabling effective data visualization to foster a culture of data use for decision-making. Uganda has been utilizing District Health Information Software 2 (DHIS2) as the main Health Management Information System (HMIS) to generate data for effective policy formulation, implementation, monitoring and evaluation of health programs since August 2012. This open-source configurable platform provides tools to collect, analyse, visualize, share, and validate both individual-level and aggregate data. DHIS2 has both been used as a general platform and provided multiple tools and packages to support the effective implementation of a variety of health programs including but not limited to the Expanded Program on Immunization (EPI), HIV, tuberculosis (TB) and malaria (HTM) [4–6]. This system has helped identify gaps in immunization coverage, monitor malaria rates, track TB treatment, reduce stock wastage, optimize cold chain management, and follow up on immunization reports [5, 7]. In addition, the implementation of the DHIS2 Tracker has improved the timelines of national vaccine schedules, supported the monitoring of campaigns, provides case management, and reduced drop-out rates [4, 7].

There has been significant investment in the DHIS2 system to enhance data management, use and improve health program outcomes in Uganda. For instance, in 2020, with support from Global Fund and the US CDC (as well as PEPFAR and WHO for HIV/AIDS programs), and technical support from the Health Information System Program (HISP) Uganda, the country adopted DHIS2 as the national system for weekly reporting of TB notification data. The system also has an integrated dashboard that aggregates the data on weekly basis and automatic reports are generated for analysis. While significant investments have been made, the evidence on the effect of such systems on data quality, timeliness of reporting and decision-making is limited [8–11]. This study aimed to describe the enablers and persisting barriers to the use of DHIS2 system for supporting operational, program and policy decision making in Uganda.

## Materials and methods

Uganda has four regions and 136 districts which are further divided into sub-counties, parishes and villages. Uganda’s health care system is divided into levels/hierarchies of service provision. At the lowest level we have the primary health centres level II which are closest to communities at village level and offer basic outpatient care serving a smaller population. As the population increases and services increase, at sub county level we find health centre III, where more services are offered. In patient and surgical services are offered at higher levels at HCIV, general hospitals and referral hospitals. Health centres III and IV indicate the level at which most reporting into the DHISII starts [3].

**Figure 1:**
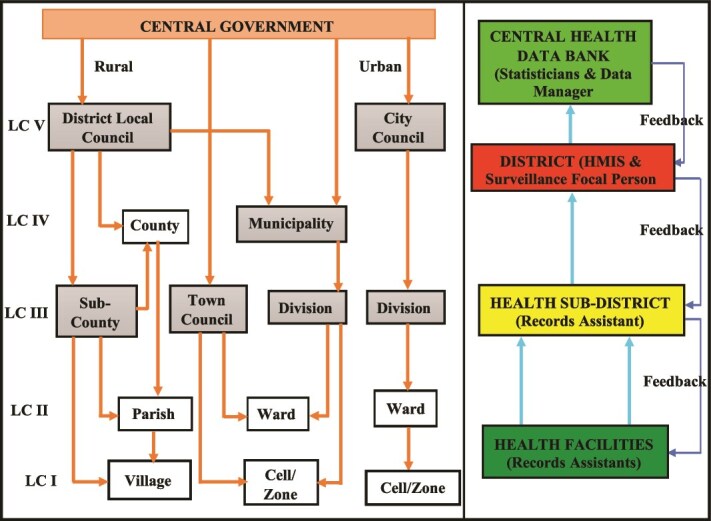
DHIS2 data flow from facility level to central health databank. LC- local council, HMIS- health management information system [12, 13].

Health records are compiled in health facilities and then submitted to the health sub-district, to the district and then to the national level at the division of health information [12, 13] (Fig. 1). A mixed-methods study was conducted in Uganda using nationwide secondary data from DHIS2 along with primary qualitative data collection in six districts.

### Secondary data analysis

#### Indicator selection

We reviewed the DHIS2 system for indicators comparable to globally validated indicators and selected three program specific indicators to measure indicator data availability: Expanded Program for Immunization reporting timeliness (%), proportion of health facilities reporting monthly and the proportion of health facilities reporting weekly as employed in a previous study done in Uganda [14]. We obtained monthly indicator data for the years 2020, 2021 and 2022 and for all districts in Uganda.

#### Analysis

After obtaining the average for each of the indicators, we split the data into tertiles based on the overall range and distribution, as illustrated in Table 1. The tertiles were based on the overall distribution of the data such that the cut offs were different for each indicator based on the range of the data. Thereafter, districts that were categorized as suboptimal performers for all three indicators were called low adopters, those categorized as medium (moderate performers) across the three categories were called medium adopters and those that were high (best performers) across the three categories were called high adopters.

**Table 1:** Results of the data categorization of the 3-year performance averages

Variable	Score^*^	Number of districts (%)	Adoption level
Average EPI - reporting timeliness (%)	56.7–82.7	48 (32.9)	Low
	82.8–91.1	49 (33.6)	Medium
	91.2–99.5	49 (33.6)	High
Average proportion of health facilities reporting monthly	30.8–33.4	48 (32.9)	Low
	33.5–41.9	49 (33.6)	Medium
	42.0–99.8	49 (33.6)	High
Average proportion of health facilities reporting weekly	43.3–79.7	48 (32.9)	Low
	80.1–90.1	49 (33.6)	Medium
	90.2–99.1	49 (33.6)	High

^*^ *Average based on proportion of health facilities reporting per district.*

### Qualitative data collection and analysis

#### Sampling approach

Two districts were purposively selected from each category based on feasibility and ability to learn from the districts about DHIS2, in consultation with stakeholders.

Informants were purposively selected to satisfy study objectives and interviews were conducted until data saturation. Qualitative interviews explored common practices of the use of the DHIS2 system, perceived enablers, barriers and effects of DHIS2 on health and health system outcomes. The interviews were conducted at the national, district and facility level. The district interviews were conducted in six districts across the DHIS2 adoption spectrum including Jinja, Buikwe, Maracha, Yumbe, Kakumiro and Budaka. The facility level interviews were conducted in 12 purposively and conveniently selected health facilities across the six districts including two hospitals, four health center IVs and six health center IIIs. A total of 58 qualitative interviews were conducted: 5 at national level, 17 at district level and 36 interviews at the health facility level. This is summarized in Table 2.

**Table 2:** Summary of the interviews conducted across the six districts

		Low performer		Medium performer		High performer		
Level		Buikwe	Jinja	Budaka	Kakumiro	Maracha	Yumbe	Total
**District**	District health Officers	1	0	1	1	1	1	5
	District Biostatisticians	1	1	1	1	1	1	6
	District planners	1	1	1	1	1	1	6
**Health Facility**	Health facility in charge	2	1	2	2	2	2	11
	Data entry clerks/HMIS focal person	2	4	2	2	2	2	14
	EPI focal person	0	0	0	0	1	2	3
	HIV focal person	1	0	1	0	1	1	4
	TB focal person	2	0	0	1	0	0	3
	Malaria focal person	0	0	1	0	0	0	1

#### Data management and analysis

Interviews were audio recorded and transcribed verbatim. A codebook based on study objectives was created with predetermined codes categorized into themes. This codebook was converted into a Microsoft Excel matrix. Finally, framework matrix analysis was conducted and data from transcripts was extracted based on the codebook into the matrix. The results were synthesized and summarized with illustrative verbatim quotes.

## Results

### Adoption of DHIS2 at sub-national level

The DHIS2 systems data analysis, conducted at the district-level, helped stratify districts according to level of adoption, as illustrated in Table 1. An average adoption score was computed based on the average of all three indicator scores listed below. The average ranged from 97% for high adopters to 60.3% for low adopters. From this, five districts were identified with a high-level of DHIS2 adoption, two districts were identified with medium adoption, and 11 districts were identified as low adoption. Two districts were selected from each of these categories.

### Enablers of using DHIS2 system

**Figure 2:**
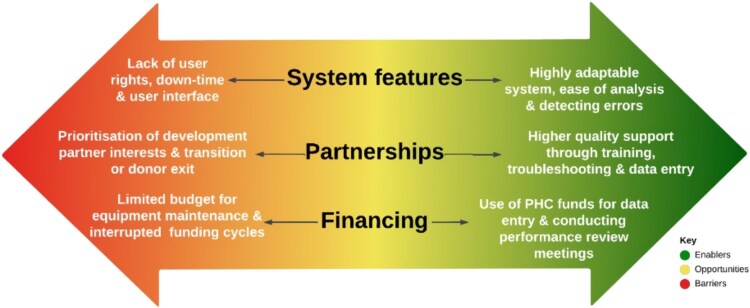
Opportunities presented by the dual role of system features, partnerships and financing in the adoption and use of DHIS2 system in Uganda.

A number of factors were highlighted as facilitating the adoption and use of DHIS2 in Uganda. These are summarized in Table 3 and Fig. 2 below.

**Table 3:** Enablers for the adoption and use of DHIS2 system in Uganda

Enablers	Sub themes
Enabling infrastructure	Access to internet connectivity
	Availability of appropriate hardware (computers, phones)
	Stable power source (generators, solar energy)
	Availability of data collection tools (HMIS forms, registers, stationary)
User interface and system features	Ease of use (data analysis, interpretation)
	Reliability and easy accessibility
	Ability to detect errors and gaps in data
	New innovations and apps (Smart Paper Technology)
	Compatibility with different hardware
	System generated reminders
	Facilitates quick feedback on performance
Training, support and interpersonal reasons	Experience with other digital tools and tech-savviness
	Trainings and mentorship by MoH and implementing partners
	Commitment towards job and improved health outcomes of district
	Continuous medical education initiatives
Partnerships	Technical support through training, mentorships and troubleshooting
	Data entry support
Policy and legislation	Policy mandate to report
	Demand from supervisors and partners for outputs/deliverables
Incentives	Financial incentives based on higher performance*
	Non-financial incentives by MoH as recognition
	Performance review meetings motivate completion of work*
Financing	Use of Primary Health Care (PHC) budgets to finance data entry and review meetings
	*For high performing districts

#### Enabling infrastructure

The availability of supportive infrastructure was highlighted as a key enabler for the adoption and use of the DHIS2 system. These included internet and network, as well as hardware such as computers and phones that eased data entry and visualization. Some noted the availability of power connectivity with backup systems such as generators and solar systems imperative to the use of DHIS2, as indicated in the quote below:

*“Power availability is a key enabler and even when power is off, they put a generator on the last 3 months, we didn’t have any power issues that facilitated my reporting”* (TB focal person, low adopter district)

*“We have been able to install a solar system because the internet and power moves together. However, we have also the hydroelectricity power and is on and off, that why we have solar as backup”* (Health facility in-charge, high adopter district)

#### User interface and features

The system’s user-friendly attributes allow for quick and easy data entry, analysis and visualization. The system can also be accessed at any time, and its ability to detect errors enabled the use of DHIS2. Additionally, new innovations within the system, such as paper smart technology and dashboards have facilitated the increased use. For instance, some of the key informants reported:

*“I like DHIS2 because it is versatile and now those new innovations which they have added are doing us a good service."* (HMIS focal person, moderate adopter district)

*“DHIS2 is now more user friendly compared to when I started working. It makes analysis easy and helps you know where you are because it gives timely feedback. You will know how a district or facility is performing in all disease programs. It also has a number of parameters.”* (District biostatistician, low adopter district)

Some key informants also reported that having access rights and passwords enabled access and use of DHIS2 system and data for planning, as emphasized below:

*“The different players/stakeholders are given rights to access this system and when you access it at management level, you can really see the facilities which are reporting, and those reporting on time as well as the facilities which tend to have errors in their data.”* (District Health Officer, low adopter district)

#### Training, support and interpersonal skills

Key informants reported that having the capacity and knowledge to use the system was an efficient contributor to the adoption and use of the system. DHIS2 systems were enabled by having several information assistants who supported data entry. The providers also noted staff commitment and teamwork as key enablers for using the system.

*“I think having two information assistants facilitates the use of DHIS2. We have two of them. And so, in the absence of the other, one can back up.”* (Health facility in-charge, high adopter district)

#### Partnerships

The implementing partners provided technical, capacity-building and financial support, which was reported as an enabling factor. For instance, in Jinja District, implementing partners set up a data center and recruited data entry clerks to support data entry. Implementing partners have also supported the training of staff in data entry and analysis. HISP Uganda also offers technical support in troubleshooting any challenges within the system. The IPs also provide computers and phones to support data entry. These are emphasized by the quotes below:

*“Implementing Partners (IPs) support us with data cleaning to improve data utilization at source and by doing analysis, they support us to analyze our data."* (District Health officer, low adopter)

#### Policy and legislation

Regarding motivation for reporting, most of the key informants noted that reporting was their mandate, obligation and a required output as part of the work they do.

*“it is your output so if you have been working, where is your output so I think there will be questions if you really worked especially when you delay reporting so it is my mandate to report data on a timely basis."* (Health facility in-charge, low adopter district)

#### Incentives

A number of key informants were motivated to report by the provision of non-financial incentives such as recognition for performance, and therefore used the system to monitor their performance and take needful action. Routine data review meetings held regularly to assess performance also compelled many to report. This was emphasized below:

*"The performance reviews push us to report. We also have reviews at facility level before the quarterly review meetings at the district happen".* (Health facility in-charge, high adopter district)

The availability of financial incentives for entering data and submitting reports on time and an earmarked line item in the Primary Health Care budget for purchasing cellular data and digitization equipment were also reported as key enablers.

*“The in-charge motivates me with facilitation to submit the reports, buying data for instance, I need data when submitting a maternal death report by connecting my phone to the computer and sometimes I use part of my little salary because this is my role.”* (HMIS focal person, medium adopter district)

### Barriers to the use of DHIS2 system

A number of factors were highlighted as barriers to the adoption and use of the DHIS2 system in Uganda. These are summarized in Table 4 and Fig. 2.

**Table 4:** Barriers to the adoption and use of DHIS2 system

Barriers	Sub themes
Infrastructural inadequacies	Poor internet connectivity and network
	Unstable power and backup systems
	Stock out of tools, registers
	Obsolete hardware
Unsupportive system features	Prolonged system down-time
	Lack of systems user rights, access and login credentials
	Uncoordinated systems updates
	Non-intuitive user interface due to increased complexity following systems optimization to incorporate additional conditions
	Data loss due to power and back-up
	Data entry mismatch
Human resource challenges	Insufficient staffing levels
	Work overload and suboptimal remuneration
	Limited workforce capacity to use technology
	Data quality/integrity concerns with collection, entry and reporting
	Lack of training for new tools
	High staff turnover in private facilities
	Limited on-the-job mentorship
Complex data entry protocols	Cumbersome system with multiple steps for reporting
	Time-consuming
	Dual burden of data collection (paper, digital)
	Non-streamlined data entry with many people at different stages
Dependence on external agencies	Prioritization of implementation partners’ interests
	Dependence on partners for support instead of transition of capacity
Financial constraints	Lack of budget for equipment maintenance
	Funding cycles interrupt internet connectivity

#### Equipment and infrastructural inadequacies

Participants highlighted several infrastructural challenges affecting the use of DHIS2 including unstable power supply, poor internet connectivity and network especially in the rural areas which affected the use, delayed data entry and reporting. This was emphasized by one the key informants below:

*"The internet is sometimes off. Unstable power yet there is no power backup, unstable network of the system for instance, last month as I was entering data, all the information disappeared from the system.”* (Health facility in-charge, medium adopter district)

The stock out of data registers forced facilities to improvise forms for data capture, further compromising the data quality. This was emphasized by one of the key informants.

*“Stock-out of the reporting tools can also sometimes affect data reception. For instance, you may be expecting a report but the in-charge at a given facility will inform you that he/she does not have a tool in place."* (District Biostatistician, low adopter district)

The lower health facilities lack computers, and hence cannot enter the data directly into DHIS2 systems. They must travel to the district and submit the reports in hard copies for entry. This is exacerbated by lack of transport, which means that the reports are received late, and hence delayed reporting.

#### Unsupportive system features

Respondents also reported barriers related to the DHIS2 system including prolonged down time, uncoordinated systems updates, difficult user interface, data loss when power goes off and restricted access. Some respondents reported the lack of access to the system especially for the health facility in-charges and district planners which delayed access to data for planning and assessing the quality of data entered into the system. Some noted that DHIS2 can only be accessed by the district statisticians, implementing partners and selected data entry clerks.

*“There is still a challenge of DHIS2 being restricted to a few people and it is limited to high volume areas and how I wish that this service could be extended to all the other programs and private facilities because they have data that the country is missing."* (Health facility in-charge, low adopter district)

Some respondents also reported that they are aware of the reporting dates, and that the system gives less time to validate what was entered into the systems, hence affecting data quality, as emphasized below:

*"The demotivation is that we are given a short period of time to change (actually, there is no time for you to change what has already been submitted) and also people are not aware of that time period allowed for submitting the reports. The district people give us a deadline by which if our data is not submitted, we risk not having our data in the system."* (HMIS focal person, high adopter district)

#### Complex data entry protocols

Some of the participants reported that the system was complex and cumbersome to use, and the data entry forms were lengthy, especially for HIV. Paper-based data collection complicates data entry and affects the quality of the data. It was also noted that the many registers used for data collection and the many people who collect data from the different departments and different stages complicate the use of the system. Some health facilities do not enter data directly at the health facility level, which contributes to delays in reporting. These are emphasized below:

*"Lengthy tools for data entry-""The entire nutrition and HTS template is quite long and so, sometimes you have to go back. If they were a bit smaller to see the matching edges. Sometimes there is no power thus hinders timely reporting."* (HMIS focal person, low adopter district)

#### Human resource challenges

Several human resource constraints on the use of the DHIS2 system were raised. These included inadequate staffing levels of health information and HMIS focal persons in health facilities, coupled with work overload and suboptimal remuneration, which affect the quality of data. Some participants also reported the limited capacity to use computers and, later on, the system. Private facilities were affected by high staff turnover among the few trained staff.

*“….another challenge at district level is we do not have enough human resources since we at times rely on people to support us such as the volunteers, students and the like, who normally leave us, and this affects timely reporting."* (Health facility in-charge, medium adopter district)

#### Dependence on external agencies

It was reported that the systems depend a lot on the implementing partners who, upon their exit, render the districts powerless and demotivated. For instance, in Jinja, the district has no control over the systems, all activities are planned and determined by the implementing partners as expressed by some of the participants below:

*"Sometimes you look at their resource envelope to plan for the activities and at times they plan for you for instance, they can say that we are doing a verification in this and since their activities are partner led then you have to rely on them and work on their schedule. They can support a program in a particular period and later state that they are no longer supporting it so such dynamics affect the process in terms of data timelines and verification."* (District biostatistician, low adopter district)

In Jinja, where data entry is entirely supported by the implementing partners, respondents reported cases where some health facility data does not get reflected in the system and yet the facility submitted the data for entry. This was attributed to work overload, and some data is not entered into the system. One health facility respondent reported:

*“Sometimes the data submitted to the district is not entered by the clerks at the district. The challenge is that we are submitting data but some of it is not being entered into the system.”* (Health facility in charge, low adopter district)

#### Financial constraints

Respondents reported a limited budget for equipment maintenance, capacity building, and motivating staff. Some facilities that budgeted for internet data bundles reported that they are affected by the funding cycles. Consequently they sometimes run out of internet data bundles and they must wait for another funding cycle, which affects timely reporting.

*“I should be having the internet to enable me and when the bundles given are finished, yet at times the money has not yet come, you have to improvise means to make sure you report so you end up incurring the expense to make sure you report.”* (HMIS focal person, high adopter district)

## Discussion

DHIS2 remains the world’s largest HMIS platform used in more than 100 countries. The system is used by ministries of health in more than 80 low and middle-income countries, capturing information for more than 3.2 billion people (40% of the world’s population) [6, 15]. Like many countries where the system is used, the DHIS2 was rapidly scaled up in Uganda, but its use has varied across districts with some consistently performing well and while others perform less optimally [15–17]. Our in-depth analysis reveals several reasons for this and juxtaposes the facilitators of DHIS2 adoption with the barriers.

Country ownership of DHIS2 has been critical to the widespread use of the system. Uganda has mandated the use of DHIS2 as the reporting platform for health data and this has enhanced reporting completeness by districts into the system, most especially for the government owned facilities [18]. Like many LMICs with pluralistic health systems, the private sector plays a major role in service delivery, accounting for more than 50% of services delivered [19]. However most private health providers in Uganda have invested in health information systems that focus on simplifying their administrative activities rather than collecting performance data. Even though the private sector in Uganda is required to use DHIS2, with a few exceptions, their capacity building and ultimately adoption of the DHIS2 system has been less optimal and inconsistent across districts as reported elsewhere [20]. This sub-optimal adoption of the system by the private sector is also partly driven by the lack of inter-operability between their existing information systems applications and the DHIS2 system. In order to enhance private sector adoption of DHIS2, government’s investments should go beyond merely demanding data reporting compliance from the private sector, to providing leadership and governance, co-creating harmonized information systems, as well as regulatory oversight by engaging the private sector as a co-investor in the public health systems.

Considerable investments have been made to expand the coverage of critical infrastructure necessary for the adoption of digital tools. These investments have enhanced internet connectivity, the availability of computers and phones, and the provision of stable power sources and/or useable alternatives such as generators and solar panels. Internet connectivity emerged as both an enabler and a barrier in our study. This is because stable internet cannot be guaranteed at any facility and network fluctuation is experienced across the country. The ability to access the system is facilitated when the network is good and barriers are experienced whenever the network is poor. Our study did not assess strength of connectivity across districts but relied on responses from interviewees because they experience both network connectivity extremes.

The Government of Uganda has demonstrated commitment towards the effective realization of its digital health strategy through making some investments in infrastructure [21]. These have included, but are not limited to the provision of funding for the alert system via telecom providers, as well as enhancing electrification in hard-to-reach areas. According to Kinkade (2022), investments in DHIS2 enabled Sri Lanka, Sierra Leone and Uganda to quickly adapt their DHIS2 systems during the COVID 19 pandemic to cater for the demand for quick data for decision making [22]. However, infrastructure gaps persist in the form of outdated or broken computers, poor internet connectivity in some geographies, unstable power and faulty power backup systems as well as the stock out of registers. These persisting barriers underpin the need to continuously monitor, maintain, repair and update electronic software and hardware across the country including identifying opportunities to utilize existing telecom network providers to subsidize costs for DHIS2 for assured connectivity.

Over the past decade, the use of the DHIS2 system has been expanded globally and the system has been optimized and adapted to country and even program needs. This open-source platform has been adapted to include data validation, visualization and analysis tools, which enable easy access and manipulation of health data at both national and subnational levels [6]. Moreover, the increased adoption of electronic forms versus the traditional paper-based formats, has facilitated built-in data quality control measures. Among others, the system was lauded for its ability to ease data analysis and interpretation, and because of its interoperability with mobile data collection platforms, can be easily accessed at any time [23]. This level of systems optimization has enabled decision makers to have reliable and easily interpretable data at the click of a button. However, the bid to optimize the system has been associated with an increasingly complex data entry workflows resulting in user dissatisfaction. Further, multiple health outcomes are reported in DHIS2 and these often have different workflows that prioritize implementing partner objectives for data reporting. Such complexity in data entry coupled with disharmonious systems and difficulty in managing new interfaces disrupt the user experience. In addition, as the system continues to expand reach, stringent measures have been institutionalized to enhance data security, locking out some users. Consequently, the users of the DHIS2 system in Uganda expressed discontent with the lack of systems user rights, login credentials and limited access to some of its features due to enhanced data security which constrains their ability to use the system effectively.

Significant proportion of total DHIS2 investments have been committed to training personnel in using these systems. Training modules have included data entry, analyses and visualization among others. This has increased the number of skilled staff available, enhancing the capacity of facilities and districts to enter and analyze data in a timely manner [17]. However, the respondents noted that where staff shortages exist, the additional responsibility to enter data without corresponding remuneration led to perceived work overload as well as quality or integrity concerns with data practices. In private facilities where staff tenure is not permanent, it was reported that staff turnover frequently led to loss of capacity, even where personnel had been trained, consequently compromising capacity to use the system. Workers also highlight the lack of refresher training to update staff whenever new tools or indicators were introduced. Furthermore, in some settings, respondents opined that training should not be restricted to information personnel (data entrants, focal persons), but rather training should be made accessible to all cadre so that all human resource for health can participate in data generation and use.

In Uganda, like many other countries where DHIS2 is in use, government’s budgetary support to information systems has remained minimal. Estimates of the government health expenditure allocated to information systems in most of other African countries such as Nigeria and South Africa are reported to account for <1% of the total health expenditure [24]. Since 2016 development partners with WHO technical advice and the support of the Global Fund, migrated to DHIS2 as the national reporting platform for three national programs (HIV, TB and Malaria) and these have continued to date. With development partner support other countries have adapted the DHIS2 to include modules of entomology and vector control in order to support countries to improve the collection and use of entomological and vector control interventions data and its use to inform programmatic decisions [25]. Ultimately, development partner support for DHIS2 has been the lynchpin for facilitating the adoption and optimization of DHIS2 [16]. For high performing districts, implementing partners were highlighted as providing support for capacity building, mentorship and infrastructure. Development partners also integrated interventions like performance based monitoring and financing (also known as results based financing (RBF), which requires monitoring performance data via the DHIS2 system [18, 26]. In Nigeria the implementation of RBF using the DHIS2 system enhanced data robustness, timeliness and reliability [26] and our study reports similarly. High performing districts were characterized by enhanced partner support such as the implementation of performance-based initiatives which boosted data management and consequently the consistent use of DHIS2. Financial support for the system enabled providers at the frontline to provide financial incentives for data management, but oftentimes the delays in funding cycles and the minimal funds provided did not allow for equipment maintenance compromised DHIS2 systems adoption. However, this has also resulted in dependence on external agencies for supporting DHIS2, blighting the development of sustainable funding mechanisms for health information systems and threatening implementation whenever partners withdrew funding. With the current changes in global health financing climate, governments need to carefully and expeditiously formulate a transition plan for sustaining their information systems with reduced reliance on external funders.

Our findings offer several specific recommendations to optimize DHIS2 use and adoption in Uganda. Firstly, the capacity for HRH should be enhanced through needs-based training across higher and lower cadres, and include a wider selection of skills ranging from basic IT functions to specific DHIS2 functions. Local ownership should be prioritized by reducing dependence on donor funding, decentralizing decision-making to facility-level teams, and transitioning from external support to sustainable financing. Routine maintenance of equipment and supplies should be institutionalized as regular practice at the facility level. Further, efforts to streamline workflows, debulk lengthy data entry formats and remove redundancy in indicators should be pursued actively. To shrink the growing digital divide in lower middle-income countries, attention should also be given to improving DHIS2 adoption in low-connectivity areas and among lower-skilled staff, ensuring equitable access and use. Given the rapid expansion of the digital health ecosystem often driven by implementation partner support, planning for sustainability must be integral from the outset, even when partners initiate certain services.

The strength of this study is that it utilizes existing DHIS2 data to purposively select the study district on the basis of their performance. This enhances our understanding of how DHIS2 may or may not work well with in different settings across the country. The limitation is that findings from these districts may not necessarily be generalized to other districts in the country because of contextual differences.

## Conclusion

In conclusion, the adoption of the DHIS2 system in Uganda has advanced significantly in terms of infrastructural investments and personnel which has been beneficial for the country’s health information system, facilitating the move from unreliable, delayed paper-based reporting to high quality electronic data generation. Enhanced data availability and use for operational, program and policy decisions has led to improvements in health systems efficiency and health outcomes reported across the country. It is important to note that systems like DHIS2 require more than one-off capital investments to set up the system. We recommend training at all levels especially whenever the system updated, routinely auditing and maintaining equipment, streamlining indicators and workflows and planning for sustainability even when implementation partners are involved in the initial roll out of services. Districts with low adoption would benefit from intensified capacity building and incentives especially for private sector providers.

## Supplementary Material

Supplementary_materials_oqag002

## Data Availability

The secondary data used in this article were obtained from Uganda’s national DHIS2 system backend. The derived aggregated dataset underlying the results in this research, as well as the anonymized qualitative data will be shared on reasonable request to the corresponding author.
