# Supplementary material for: Barriers and facilitators of the effective use of DHIS2 data to improve program planning and monitoring in Uganda: a sequential mixed methods study
Source: Oxf Open Digit Health. 2026 Jan 14;4:oqag002. doi: 10.1093/oodh/oqag002 (PMC12870114; doi:10.1093/oodh/oqag002)
Supplement: Supplementary_materials_oqag002 [file supplementary_materials_oqag002.docx]

**Supplementary Files**

**Supplementary Table 1. Details about the selected districts**

| ***District*** | ***Region*** | ***Population*** | ***Health facilities*** | ***Adoption level*** |
| --- | --- | --- | --- | --- |
| ***Maracha*** | ***West Nile*** | 219,500 | 19 health facilities: 16 public, 2 private not for profit (PNFP), and 1 private for profit (PFP) | ***High*** |
| ***Yumbe*** | ***West Nile*** | ***99,794*** | 37 health facilities: 27 public health facilities, 3 PNFPs, and 7 PFPs | ***High*** |
| ***Kakumiro*** | ***Western*** | 473,400 | 32 health facilities; 14 public health facilities, 17 PNFPs, and 1 PFP | ***Medium*** |
| ***Budaka*** | ***Eastern*** | 29,100 | 21 health facilities: 16 public, 2 PNFPs, and 3 private for profit | ***Medium*** |
| ***Buikwe*** | ***Central*** | 422,771 | 56 health facilities: 29 public, 23 PNFPs, and 4 PFPs | ***Low*** |
| ***Jinja*** | ***Eastern*** |  | 85 health facilities: 50 public health facilities, 21 PNFPs, and 14 PFPs | ***Low*** |


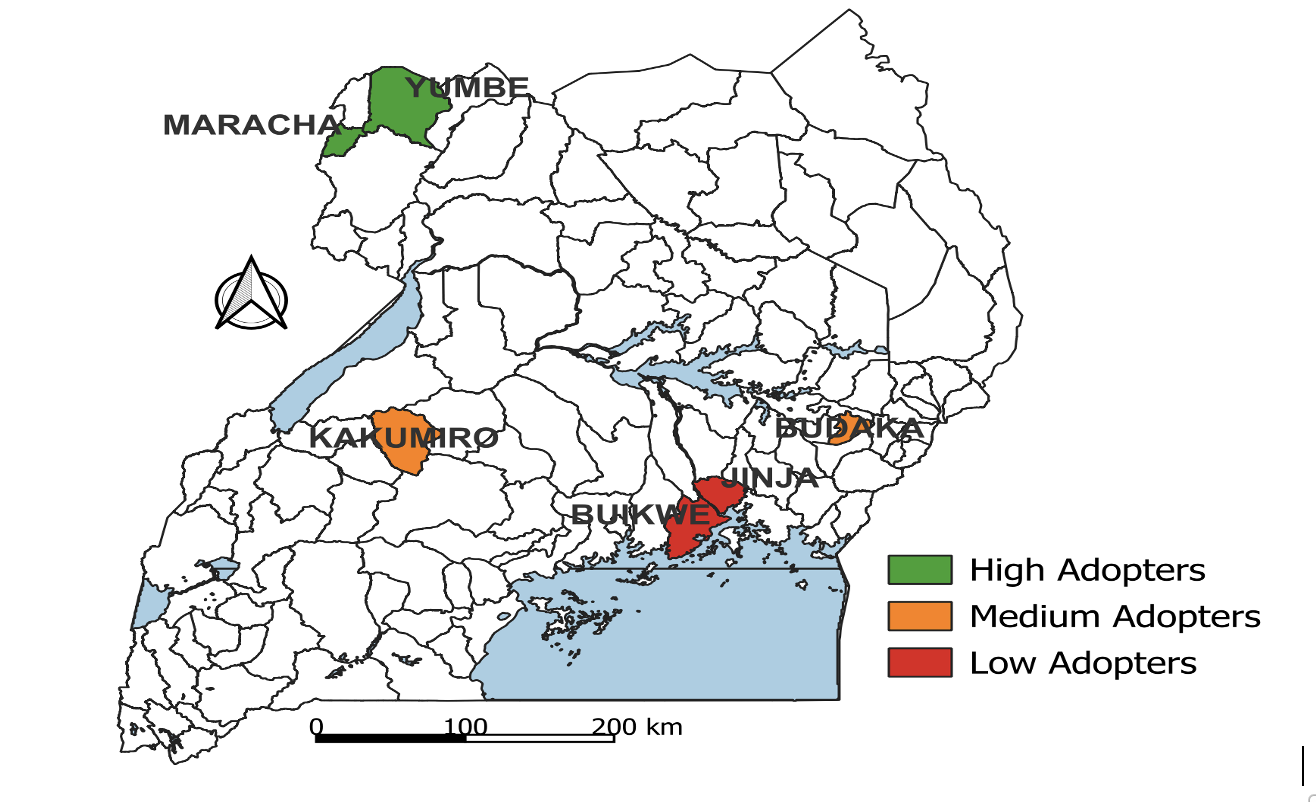


**Supplementary Figure 1. Map showing the selected districts for the study**
